# 

**Published:** 1871-06

**Authors:** 


### BOOK NOTICES.

"The Life of Christ," 8vo, 861 pages; handsomely bound in muslin; issued by the American Tract Society, 150 Nassau Street, New York; written by the Rev. WILLIAM HANNA, D. D., LL. D., author of the "Life of Rev. Dr. Chalmers," after a personal visit to Palestine to examine the localities and gather all the information possible, which could in any way be made use of to enable him to handle the subject truthfully and lucidly. The book is printed in large type, and on beautiful white paper. It is just such a book as any loving Christian can read over and over again for a lifetime with pleasure and profit.

"Desk and Debit." A beautiful little 12mo of 334 pages, with fourteen illustrations, from the irrepressible press of Lee & Shepard, Boston, and Lee, Shepard, & Dillingham, New York. This volume of OLIVER OPTIC describes in a very enchanting way to boys the "Catastrophies of a Clerk," and if it persuades one young man to determine never to go in debt, OLIVER OPTIC will not have lived in vain. Debt, the fruitful source of the fell destroyer, carking care; of mortification; of freedom, of independence, of manliness; debt, which makes a man an abject, cringing slave, introducing him into the practice of inventions and equivocations and subterfuges which degrade the whole moral nature.

"Bible Biography; or, the Lives and Characters of the Principal Personages recorded in the Sacred Scriptures," with an Introduction by HENRY WARD BEECHER, and an Appendix containing thirty dissertations on the Evidences of Divine Revelation, being a complete summary of Biblical Knowledge. Embellished with nearly three hundred engravings. Sold only by subscription, and published by Lee & Shepard for W. L. Goss & Co., No. 68 Cornhill, Boston. This is a handsome 8vo volume of 491 pages, and contains a vast amount of information explanatory of Bible subjects, and making clear its meaning. It is difficult to overestimate the value of a book of this kind in Christian families, and they are fortunate who feel able to purchase it.

"Galaxy," published monthly at $4.00 a year, by Sheldon & Co., 677 Broadway, New York, prints four times as many copies to meet the public demands as it did under its previous management. Every number contains a vast amount of entertaining and instructive reading, its contributors being among the very best writers in the country. Its increasing popularity is well merited by the energy and enterprise of its present management.

"Zell's Popular Encyclopædia: a Universal Dictionary of English Language, Science, Literature, and Art." By L. COLANGE, LL. D. Vol. 2 and last, from I to Z. Philadelphia. Illustrated by over five hundred wood-cuts. For completeness, succinctness, and reliability, it is the most useful encyclopædia of its kind in the English language, bringing as it does, the results of progress, discovery, and improvement down to the present year.

